# Mistrust and misinformation in a colorectal cancer care: rebuilding credibility in the digital age

**DOI:** 10.1007/s10151-026-03307-7

**Published:** 2026-04-10

**Authors:** Ankita Kulkarni, Nikhil Pawa

**Affiliations:** 1 Department of Bioengineering, Imperial College London, London, UK; 2 Colorectal and General Surgery, West Middlesex University Hospital, Chelsea and Westminster NHS FT, Twickenham Rd, Isleworth, TW7 6AF UK

**Keywords:** Colorectal cancer, Medical mistrust, Health misinformation, Cancer screening, Social media, Digital health communication

## Abstract

Mistrust in healthcare and the rapid spread of misinformation have become critical challenges in contemporary colorectal cancer (CRC) care. While diagnostic and therapeutic strategies have advanced, many patients now arrive at clinical appointments influenced by online narratives that distort risk, undermine screening, and question the intentions of clinicians and health systems. These dynamics are particularly evident among younger adults, who represent a growing proportion of CRC diagnoses and often rely on social media as a primary source of health information. This commentary explores how mistrust and misinformation intersect across the CRC continuum, from symptom interpretation and screening decisions to treatment acceptance and survivorship. It highlights how emotionally persuasive digital content can overshadow evidence-based guidance and complicate shared decision-making and delaying timely care. Addressing these challenges requires a deliberate shift in clinical communication: acknowledging uncertainty, engaging with cultural and digital contexts, and adopting a proactive presence in public information spaces. Strengthening trust is now inseparable from delivering high-quality CRC care and represents a collective responsibility for clinicians, institutions, and professional societies.

Colorectal cancer (CRC) care has entered an era of unprecedented innovation. Yet, progress in prevention and treatment has been paralleled by a quieter, slower challenge: the erosion of public trust and the unchecked spread of misinformation. Over the past decade, CRC incidence has risen markedly among adults under 50, with early-onset CRC (EOCRC) increasing by approximately 1–2% per year worldwide [1] and now accounting for 18–20% of rectal cancer diagnoses in some regions [1, 2]. Younger patients are more likely to present with stage III–IV disease, often after 4–6 months of symptomatic delay prior to first clinical assessment [3]. Against this backdrop, clinicians increasingly meet patients whose understanding of symptoms, screening, and treatment is shaped less by clinical guidance and more by online narratives. Navigating this evolving landscape demands that colorectal specialists not only provide clinical excellence but also reclaim credibility in an information-saturated world.

## The anatomy of mistrust

Trust remains central to every aspect of cancer care, from screening participation to adherence with multimodal therapy. Medical mistrust is defined as a lack of confidence that healthcare providers, institutions, or scientific authorities will act in a patient’s best interest [4, 8]. Across population studies, high medical mistrust is associated with an up to 50% reduction in CRC screening uptake [4–6], lower colonoscopy completion after a positive stool test [4], and significantly reduced chemotherapy initiation even when clinically indicated [7]. Patients with elevated mistrust are also more likely to disengage from perioperative or surveillance pathways [4–6]. These effects are particularly pronounced in historically underserved populations, reflecting cumulative experiences of structural inequity and communication failure.

Today, mistrust is no longer confined to individuals. It has migrated online, amplified by social media algorithms that reward sensationalism over accuracy, with false narratives accumulating credibility through repetition and shaping patient perceptions long before they enter the clinic. The impact is especially marked among younger adults, who increasingly bypass traditional sources of clinical guidance in favour of social media content, placing the fastest-growing CRC population at the greatest risk of misinformation [14].

Misinformation, often shared without malicious intent, thrives in these digital environments. Emotional narratives and anecdotal “cure stories” frequently overshadow empirical data, giving traction to claims that conventional treatment is unsafe, unnecessary, or profit-driven. Analyses of cancer-related social-media content show that 18–20% of posts explicitly express mistrust toward clinicians or health systems [6], eroding confidence in evidence-based care and forcing clinicians to counter deeply embedded beliefs rather than isolated misconceptions.

## Screening: where misinformation strikes first

CRC screening illustrates the intersection of mistrust, misinformation, and measurable harm. Population-based screening programmes reduce CRC mortality by 15–30% [10], yet participation remains inconsistent. Online content frequently misrepresents colonoscopy, exaggerating discomfort or overstating complication risks [11], while stool-based tests are dismissed as inaccurate despite robust performance within organised programmes [12]. In parallel, alternative health narratives promote restrictive diets, colon cleanses, or herbal regimens that promise prevention but lack evidence.

These messages appeal to autonomy and identity, often resonating more strongly than public health campaigns based on statistics. In contrast, community health-advisor programmes, culturally aligned outreach, and survivor-led initiatives have demonstrated 10–20% absolute increases in screening uptake [9, 10]. Within consultations, mistrust can be mitigated through transparency, acknowledgement of uncertainty, and invitation of questions; trust is built not by information volume but by clarity, empathy, and consistency.

## The digital pseudoscience ecosystem

The scale of digital misinformation now demands a strategic response. Peer-reviewed analyses demonstrate that 20–40% of cancer-related online content contains misinformation [11, 14]; critically, false content receives up to 2.3 times more engagement than accurate posts [11]. Disclaimers such as “this is not medical advice” offer little protection against emotionally framed and repeatedly reinforced messaging [13].

Many of these narratives are further sustained by financial incentives, with influencers and alternative-therapy providers profiting from supplement sales, detox regimens, or privately marketed interventions, creating an ecosystem where misinformation is not only widespread but economically rewarded. The same algorithms, however, can disseminate credible education if clinicians and professional societies choose to engage. Respectful, visible participation is essential; dismissive rebuttals risk entrenching scepticism rather than correcting it.

Digital literacy is increasingly recognised as a core clinical competency. Understanding how patients search, interpret, and share health information allows us to anticipate misinformation before it shapes decisions. A patient who presents citing a viral post is not challenging authority; they are signalling an unmet informational need. Meeting that need is now part of contemporary colorectal practice.

## Bridging evidence and belief

Between digital misinformation and clinical reality lies a fragile space that directly influences surgical and oncological decision-making. Misinformed patients may request interventions unsupported by evidence or reject multidisciplinary recommendations. Across oncology, studies indicate that misinformation contributes to treatment delay in up to one-third of patients [15, 16]. Some postpone planned resections to pursue dietary regimens; others decline adjuvant chemotherapy after exposure to anti-treatment narratives; some travel abroad for unproven “metabolic treatments”. These behaviours arise from fear and mistrust, complicating the balance between evidence-based recommendations and patient autonomy.

In this context, preoperative communication has become a form of operative preparation: its precision and clarity are now as critical as those required in theatre.

## Treatment, decision-making, and survivorship

Mistrust and misinformation do not end at diagnosis. They influence treatment decisions, delay surgery or chemoradiotherapy, and complicate survivorship. Patients exposed to persuasive but inaccurate testimonies online may reject standard care or pursue unproven alternatives. These are not rare deviations but emerging, documented patterns [14, 16].

## Sociocultural dimensions

Mistrust is inseparable from social and cultural context. Language barriers, low health literacy, and cultural beliefs about illness shape interpretation of medical advice. Stigma surrounding bowel symptoms, fatalistic attitudes about cancer, or modesty concerns regarding screening can impede early engagement.

Health systems must integrate culturally responsive approaches: multilingual information, community navigators, trusted faith-based partnerships, and tailored public messaging. Clinician training in cultural competence and empathetic communication is no longer optional, but fundamental to equitable colorectal care.

## Rebuilding trust: a collective responsibility

Rebuilding trust requires coordinated action across clinical, institutional, and public domains. Transparent and empathetic communication strengthens credibility; consistency and equity in care delivery reinforce it. Clinicians and professional bodies must engage proactively in the digital spaces where misinformation circulates; silence is not neutral, but permissive.

Scientific progress has extended survival in colorectal cancer, but survival alone is not success if patients fear the systems that deliver it. The responsibility to counter misinformation and rebuild confidence is not an adjunct to surgical care, but part of our collective duty.

Ultimately, the future of colorectal cancer management will depend not only on what we offer but also on whether patients feel secure enough to accept it.

## Data Availability

No datasets were generated or analysed during the current study.

## Declarations

### Presentations

This manuscript has not been presented previously and is not scheduled for presentation at any meeting.
